# Editorial: Preventing psychosis in low resources settings - insights, specificities, and recommendations to the study of clinical high risk for psychosis (CHR) in low-and-middle-income countries

**DOI:** 10.3389/fpsyt.2024.1486496

**Published:** 2024-09-06

**Authors:** Feten Fekih-Romdhane, Alexandre Andrade Loch, Souheil Hallit

**Affiliations:** ^1^ The Tunisian Center of Early Intervention in Psychosis, Department of psychiatry “Ibn Omrane”, Razi Hospital, Manouba, Tunisia; ^2^ Tunis El Manar University, Faculty of Medicine of Tunis, Tunis, Tunisia; ^3^ Laboratorio de Neurociencias (LIM 27), Instituto de Psiquiatria, Hospital das Clinicas HCFMUSP, Faculdade de Medicina, Universidade de Sao Paulo, Sao Paulo, Brazil; ^4^ Instituto Nacional de Biomarcadores em Neuropsiquiatria (INBION), Conselho Nacional de Desenvolvimento Cientifico e Tecnológico, Sao Paulo, Brazil; ^5^ School of Medicine and Medical Sciences, Holy Spirit University of Kaslik, Jounieh, Lebanon; ^6^ Psychology Department, College of Humanities, Effat University, Jeddah, Saudi Arabia; ^7^ Applied Science Research Center, Applied Science Private University, Amman, Jordan

**Keywords:** clinical high risk for psychosis (CHR), low-and-middle-income countries, prevention, psychosis, psychotic disorder

Despite its low prevalence, schizophrenia represents a significant public health concern because it carries a substantial and increasing burden of disease, particularly in low-and-middle-income-countries (LAMIC) (1). The onset of schizophrenia typically occurs in adolescence or emerging adulthood, substantially disrupting the development and functioning. Over half of the individuals diagnosed with first episode psychosis (FEP) struggle to reach expected developmental milestones or to return to their premorbid level of functioning, especially those from middle-income nations and non-European countries as compared to patients from high-income countries (HIC) and Europe (2). Given these heavy negative implications, preventing schizophrenia and other psychotic disorders has been considered as the “most urgent moral, social, economic, public health, and scientific imperatives of our time” [(3), p 104]. As such, there have been growing attempts over the past three decades to intervene sooner in patients with FEP by detecting people at Clinical High Risk for Psychosis (CHR), with the main goal of delaying or preventing the onset of a full-threshold disorder and diminishing the harmful effects of longer duration of untreated psychosis (DUP) (4). However, the mean DUP was meta-analytically estimated to be still high throughout the world, and to be significantly longer in LAMIC (48.4 weeks) relative to HIC (41.2 weeks) (5). Besides, there is strong evidence to indicate that general population individuals from LAMIC exhibit greater levels of subclinical psychotic symptoms compared to those from HIC (6, 7), which might be due, in part, to cultural differences.

These observations emphasize the critical need to promote access to early intervention services (EIS), especially in LAMIC. At present, EIS are distributed unequally across the globe, being mostly implemented in HIC (i.e. Western Europe, 51.1%; East Asia, 17.0%; North America, 17.0%) (8). This geographical distribution mirrors healthcare disparities between LAMIC and HIC (9), with LAMIC being faced with low availability of mental health services, lack of resources, high public stigma of mental illness, and limited global health policy attention [e.g., (10)]. In addition, real-world studies on the CHR paradigm in some regions of the world have been particularly scarce so far. For instance, only a very few longitudinal cohort studies could be identified on CHR in the Middle East and North African region [e.g., Tunisia (11, 12)], or in the African continent [e.g., Kenya (13)]. Taken together, some LAMIC appear to be deficient or slow in complying with international standards of prevention and early intervention in psychosis and to be confronted with major challenges in developing and implementing EIS, stressing the urgent need to pay more clinical and research attention to this field in these countries (14).

In this context, this Research Topic proposes to address insights, specificities, and Recommendations to the Study of CHR in LAMIC, and comprises four papers. In the first paper, Loch et al. conducted a systematic review on CHR studies from LAMIC encompassing 109 studies. The vast majority of studies included (n = 101) were from upper middle-income countries, 8 studies were from middle-income countries, whereas no studies from low-income countries could be identified. The most commonly reported limitations in these studies were difficulties in enrolling subjects, following-up and gathering longitudinal data (20.8%), cross-sectional design (27.1%), and small sample size (47.9%). Other limitations described by the corresponding authors of the papers included were shortage of financial resources (66.7%), lack of involvement of participants in research (58.2%), and cultural barriers (41.7%). The majority of participating researchers (75%) believed that research on CHR needs to be carried-out in a different way in LAMIC compared to HIC, because of cultural and structural issues. Although characteristics of the studies involved in the review were similar to those observed in international research on CHR, several challenges have been pointed out, including lack of researchers, lack of research team stability, limited time for research work for the trained clinical assessment staff. Stigma appeared to be a key topic of concern in LAMIC, and might have negatively affected individuals’ willingness to participate in CHR research. Authors concluded that cultural factors and stigma should be addressed to improve pathways to care for individuals with psychosis in LAMIC. The second paper by Nieto et al. investigated the differences in the prevalence of stressful life events in CHR individuals and those non-CHR with familial high risk for psychosis from an upper-middle-income country in Latin America (Mexico), where there exists multiple barriers to early intervention in psychosis. Findings showed that the CHR group reported significantly more stressful events linked to negative academic experiences compared to the non-CHR group. Bullying experiences, lower educational level and starting to live with a partner were associated with an increased risk for CHR. Based on their results, authors suggested that as negative school experiences may be risk factors for psychosis, schools could be appropriate settings for implementing prevention and early intervention strategies in psychosis. The third study is a scoping review by Vivaldi Macho et al. on the impact of the institutional model on psychiatric patients in Chile from the 19th to 21st centuries. The specific objectives were to represent, compare, then discuss the mechanisms underscoring how psychiatry affects health systems and social development in the long term. Chile is a South American country that has recently joined the Latin American psychosis early detection programs initiative, and that is encountering many challenges in the EIS implementation process (15). The review analyzed 10 primary historical resource along with 10 primary studies, and suggested that the state should be responsible for, and a guarantor of psychiatric patients. In particular, the state was recommended to provide humanitarian and professional support to patients by strengthening the primary care system. The fourth study in this Research Topic by Chakrabarty et al. explored mental health problems and their cognitive and demographic correlates among COVID-19 survivors from India. The study’s findings indicated that COVID-19 patients exhibited significantly greater mental health issues one year after contracting the virus compared to matched healthy controls, including poorer sleep quality, more severe depression/anxiety, and more cognitive impairment.
